# Pluronic F68 block polymer, a very potent suppressor of carcinogenesis in the colon of rats and mice

**DOI:** 10.1054/bjoc.2000.1540

**Published:** 2001-01-01

**Authors:** G Parnaud, S Taché, G Peiffer, D E Corpet

**Affiliations:** Sécurité des Aliments, INRA, Ecole Nationale Vétérinaire de Toulouse, 23 ch. des Capelles, Toulouse, 31076, France

**Keywords:** chemoprevention, aberrant-crypt-foci, colon-cancer, PEG, pluronic, rat

## Abstract

Polyethylene-glycol (PEG) is a strong inhibitor of colon cancer in rats, and the most potent suppressor of aberrant crypt foci. 9 PEG-like block copolymers were tested in rodents, after an azoxymethane injection. Dietary pluronic F68 led to a 98.6% reduction in the number of aberrant crypt foci in a first rat study (*P*< 0.0001). Next 3 studies confirmed this pluronic efficacy in rats and mice. This non-toxic laxative seems roughly 5 times more potent than PEG for chemoprevention. © 2001 Cancer Research Campaign http://www.bjcancer.com


*British Journal of Cancer* (2001) **84**(1), 90–93
© 2001 Cancer Research Campaign
doi: 10.1054/ bjoc.2000.1540, available online at http://www.idealibrary.com on IDEAL®

Short Communication

### Pluronic F68 block polymer, a very potent suppressor of carcinogenesis in the colon of rats and mice

**G Parnaud, S Taché, G Peiffer and DE Corpet**

Sécurité des Aliments, INRA, Ecole Nationale Vétérinaire de Toulouse, 23 ch. des Capelles, 31076 Toulouse, France

**Summary** Polyethylene-glycol (PEG) is a strong inhibitor of colon cancer in rats, and the most potent suppressor of aberrant crypt foci. 9 PEG-like block copolymers were tested in rodents, after an azoxymethane injection. Dietary pluronic F68 led to a 98.6% reduction in the number of aberrant crypt foci in a first rat study ($P < 0.0001$). Next 3 studies confirmed this pluronic efficacy in rats and mice. This non-toxic laxative seems roughly 5 times more potent than PEG for chemoprevention. © 2001 Cancer Research Campaign http://www.bjcancer.com

**Keywords**: chemoprevention, aberrant-crypt-foci; colon-cancer; PEG; pluronic, rat

Dietary changes could prevent 70–80% of colorectal cancers. An approach to prevention could be the use of chemopreventive agents. Many agents have been tested in animal studies, and some are being tested in clinical trials. However, none has yet been shown to be potent and non-toxic enough to be given to people (Steele et al, 1998). We have previously established that a diet supplemented with an osmotic laxative suppresses an early putative step in the development of colon cancer in rats (Corpet and Parnaud, 1999). The putative step was the number of aberrant crypt foci (ACF) induced by an azoxymethane injection (Bird, 1987). The laxative was polyethylene-glycol 8000 (PEG), whose formula is H-(O-$CH_2$-$CH_2$)*n*-OH, with $n = 200$. PEG is the most potent known suppressor of ACF in rats, and it also strongly suppresses the occurrence of azoxymethane-induced cancers (Parnaud et al, 1999; Corpet et al, 2000). The mechanism by which PEG suppresses ACF and cancer is not known. PEG is used to initiate cell fusion in vitro (Lentz, 1994), and we speculated that PEG effect on membranes might play a role in chemoprevention. We thus looked for the chemopreventive effect of polymers based on the PEG backbone. These block copolymers combine hydrophile and hydrophobe blocks, and vary in their surfactant and membrane-binding properties.

### MATERIALS AND METHODS

A total number of 158 rodents were obtained from Iffa Credo (Lyon, France) at 4 weeks of age, and included in 4 sequential independent studies. Rats were housed by pair in stainless steel wire drop-bottom cages. Groups of 4 mice were housed in plastic box cages, with sawdust bedding. Light cycle and temperature were controlled (12 h/12 h and 22 ± 2˚C). Powdered AIN 76 diet (UAR, Villemoisson, France) and drinking water were provided ad libitum. After 7 days of acclimatization, each rat was given one azoxymethane i.p. injection (20 mg $kg^{-1}$ BW in saline, from Sigma Chem, St. Quentin, France). Each mouse was given 4 weekly injections (5 mg $kg^{-1}$). 7 days later, the rodents were randomly allocated to dietary groups. Animal care was in accordance with the guidelines of the European Council, and rodents were killed well before large tumours developed (UKCCCR, 1998).

In a first study, 28 male F344 rats were randomized to 5 groups: a control group of 12 rats fed the standard AIN 76 diet and 4 groups of 4 rats given the diet supplemented with 5% (w/w) of one of these agents: PEG 8000 (ICN, Orsay, France), pluronic F68 (PLU, also called poloxamer 188), polyoxyethylene 100 -stearate ester and -stearyl ether (Sigma) (Table 1). In a second study, 30 male F344 rats were randomized to 3 groups of 10 rats fed pellets of AIN76 diet, and drinking water pure or supplemented with 5% (w/v) PEG or PLU. In a third study, 60 female OF1 mice were randomized to 3 groups of 20 mice, and treated like the rats in the second study. In a fourth study, 40 female F344

**Table 1** Structure and main properties of PEG-like agents[a]

| Agent | Molecular weight[b] (dalton) | Hydrophobe (% MW), structure[c] | | Main physical property | Tested in study |
|---|---|---|---|---|---|
| PEG 8000 | 8000 | 0 | ===== | | 1–3 |
| PEG ester C18 | 4200 | 5 | ==+ | emulsifier | 1 |
| PEG ether C18 | 4200 | 5 | ==+ | emulsifier | 1 |
| PEG ether NP30 | 1400 | 15 | =+ | emulsifier | 4 |
| Pluronic F68 | 8400 | 20 | ==+== | detergent, foaming | 1–4 |
| Pluronic F127 | 12 600 | 30 | ==++== | gelling agent | 4 |
| Pluronic P85 | 4800 | 50 | =+= | detergent, foaming | 4 |
| Pluronic L64 | 3000 | 60 | =+= | detergent, o/w emulsifier | 4 |
| Pluronic L61 | 2000 | 90 | =+++= | defoaming, w/o emulsifier | 4 |

[a]Pluronic properties, according to the BASF corporation (http://www.basf.com).
[b]Polymer MW are approximate.
[c]Structure is shown with = for hydrophilic and + for hydrophobic blocs.

*Received 28 April 2000*
*Revised 14 September 2000*
*Accepted 20 September 2000*

*Correspondence to:* DE Corpet rats were randomized to 7 groups. Two groups of 10 rats were fed powdered AIN76 diet supplemented or not with 5% PLU. 5 groups of 4 rats were fed diets supplemented with 5% of one of the following pluronics: F127 (Sigma), P85, L64, L61 and a PEG nonylphenyl ether, NP30 (Synperonics from Fluka, St. Quentin, France) (Table 1). Body weights, food and water intake were monitored weekly. Fecal humidity was measured on pellets obtained directly at the anus.

30 days after the start of experimental diets, the animals were sacrificed by carbon dioxide asphyxiation. The colons were evaluated for ACF by Bird's procedure (Bird, 1987). After fixation between coded filter papers in 10% buffered formalin (Sigma), they were stained with methylene blue (0.1%) for 6 min, then the mucosal side was observed at 32 × magnification. ACF were distinguished from surrounding non-involved crypts by their slit-like opening, increased staining, size and pericryptal zone. All colons were scored blindly by a single observer.

Results were analysed for statistical significance with the test of Welch. Multiple comparisons with a single control were done with the test of Dunnet. Data are given as mean ± standard deviation. Two-sided $P$ values below 0.05 were considered significant.

### RESULTS

PLU and PEG strikingly suppressed ACF in the first study (Table 2). There were 71 times fewer ACF in PLU-fed rats than in controls (a 98.6% reduction, $P < 0.0001$), and these ACF were smaller than in controls ($P = 0.003$). The difference between PEG and PLU was not significant ($P = 0.24$), perhaps because groups were too small. A small polyp was found in an azoxymethane-injected PLU-fed rat. The treatments with PEG-ester and PEG-ether marginally decreased the number of total ACF, and the number of large ACF. All agents markedly increased the fecal weight and humidity (Table 2). No agent changed the rat body weight (Table 2) or the water and food intake (17 ± 1 ml $d^{-1}$ and 17 ± 1 g $d^{-1}$).

The second and third studies confirmed the effect of PLU in larger groups of rats and mice. Both PEG- and PLU-fed rats and mice had fewer ACF than controls (Table 3, all $P < 0.0001$). In mice, PLU treatment led to fewer ACF than PEG treatment ($P = 0.0003$). PLU-fed mice did not excreted more feces, or more moist feces, than controls. Thus, in mice, the chemoprevention afforded by PLU was not due to a laxative effect. Again, a small polyp was found in one PLU-fed rat. PLU and PEG did not change the final body weight of rats or mice (Table 3). Because mice drank relatively more water than rats, they received twice more PEG and PLU than rats (7.5 and 3.5 g $kg^{-1}$ $d^{-1}$ respectively).

In the fourth study, PLU was very potent to suppress ACF in female rats (Table 4). There was 56 times fewer ACF in PLU-fed rats than in controls ($P < 0.0001$), and 6 PLU-fed rats out of 10 had no ACF at all. Pluronic F127 halved the ACF number ($P < 0.01$), but the other pluronics produced no significant effect on ACF number (Table 4). The food and water intake were not changed by the pluronics (12 ± 2 g and 13 ± 1 ml $d^{-1}$), except L61 and L64. Indeed, rats refused to eat the diet with pluronic L61 or L64, even when, after one week, the inclusion level was lowered to 1%. This early fast may explain the reduced ACF size in these two groups. In contrast with the other three studies, PLU decreased the weight gain compared with control diet (–6%, Table 4).

### DISCUSSION

Dietary PLU strikingly suppressed ACF when administered after carcinogen treatment. To our knowledge, PLU is the only agent able to clear completely ACF from the colon of rats after an azoxymethane injection (shown here in 6 female rats out of 10). The results suggest, but do not prove, that PLU could be a more potent agent than PEG. When PLU and PEG were directly compared, the number of ACF was smaller in PLU-fed than in PEG-fed rodents (Tables 2 and 3), but the difference was significant in mice only. In addition, PLU was more potent than PEG to decrease the number of large ACF, a better carcinogenesis endpoint than total ACF (Caderni et al, 1995). To summarize our previous rat studies, a 30-day PEG treatment decreases the ACF number 6-fold (median of 6 independent studies) (Corpet and Parnaud, 1999; Parnaud et al, 1999; Corpet et al, 2000). Here, PLU treatment decreased the ACF number 31-fold (median of 4 rat studies). PLU was thus roughly 5 times more potent than PEG, which is the most potent suppressing agent in this model (Corpet and Parnaud, 1999). Two possibilities may however reduce the value of this finding, and should be studied further: (i) PLU might suppress ACF but not tumours, as suggested by the finding of an occasional polyp in 2 PLU-fed rats; (ii) PLU might suppress tumours in rodents but not in humans.

The pluronics, or poloxamers, are a family of nonionic surface active agents. They are block copolymers of hydrophobe propylene oxide sandwiched between two hydrophile ethylene oxide

**Table 2** Effect of feeding PEG-like compounds on azoxymethane-induced aberrant crypt foci (ACF) and fecal values in F344 rats given an AIN 76 diet

| Group[a] | *n* | ACF/ colon | Crypts/ ACF | Large ACF > 2 crypts[c] | Fecal weight (g/d) | Water in fresh feces (%) | Rat body weight (g) |
|---|---|---|---|---|---|---|---|
| Control | 11[d] | 53 ± 14[b] | 2.3 ± 0.2 | 17 ± 6 | 1.1 ± 0.1 | 30 ± 7 | 270 ± 14 |
| PEG 8000 | 4 | 3.7 ± 4.5* | 2.2 ± 0.6 | 1.2 ± 1.5* | 2.4 ± 0.1* | 68 ± .5* | 270 ± 8 |
| Pluronic F68 | 4 | 0.7 ± 1.0* | 1.5 ± 0.7+ | 0.2 ± 0.5* | 2.7 ± 0.1* | 64 ± .1* | 268 ± 4 |
| PEG ester C18 | 4 | 30 ± 17 | 1.9 ± 0.2 | 6.5 ± 2.5 + | 2.4 ± 0.2* | 57 ± 6* | 256 ± 15 |
| PEG ether C18 | 3[d] | 57 ± 62 | 1.9 ± 0.1 | 10 ± 11 | 2.0 ± 0.1* | 62 ± 4* | 265 ± 7 |
| ANOVA *P* | | 0.001 | 0.02 | <0.0001 | <0.0001 | <0.0001 | 0.36 |

[a]Diets were supplemented with (5% w/w) PEG 8000, Pluronic F68, PEG ester C18: polyoxyethylene 100 stearate ester, and PEG ether C18: polyoxyethylene 100 stearyl ether. [b]Mean ± SD,* $P < 0.01$ and + $P < 0.05$ by Dunnet's test, compared with control group. [c]Threshold was chosen so that no group has 0 large ACF. [d]One control rat and one PEG ether C18-fed rat died shortly after randomization.

© 2001 Cancer Research Campaign 

**Table 3** Effect of the addition of PEG or PLU in the drinking water of F344 rats and OF1 mice on azoxymethane-induced aberrant crypt foci (ACF) and fecal values

| Group[a] | *n* | ACF/ colon | Crypts/ ACF | Large ACF > 3 crypts | Fecal weight (g/d) | Water in fresh feces (%) | Body weight (g) |
|---|---|---|---|---|---|---|---|
| Rats | | | | | | | |
| Control | 10 | 81 ± 13[b] | 2.6 ± 0.2 | 17 ± 6 | 1.1 ± 0.2 | 38 ± 3 | 272 ± 10 |
| PEG 8000 | 10 | 16 ± 10* | 1.9 ± 0.4 | 1.2 ± 1.4* | 3.8 ± 0.5* | 67 ± 2* | 269 ± 7 |
| Pluronic F68 | 10 | 12 ± 07* | 1.7 ± 0.2* | 0.3 ± 0.5* | 3.5 ± 0.4* | 61 ± 4* | 266 ± 16 |
| Mice | | | | | | | |
| Control | 20 | 47 ± 20 | 1.6 ± 0.3 | 1.4 ± 1.7 | 0.4 ± 0.1[d] | 60 ± 7 | 31 ± 3 |
| PEG 8000 | 19[c] | 27 ± 16* | 1.6 ± 0.3 | 0.5 ± 1.0* | 0.5 ± 0.1 | 66 ± 3* | 32 ± 2 |
| Pluronic F68 | 20 | 11 ± 06* | 1.6 ± 0.2 | 0.2 ± 0.5* | 0.4 ± 0.1 | 64 ± 3 | 32 ± 3 |

[a]Drinking water was supplemented with 5% (w/v) PEG 8000 or Pluronic F68 for 30 days, starting 7 days after a single (rats) or 4 weekly (mice) azoxymethane injections. [b]Mean ± SD.* p < 0.01 and + p < 0.05 by Welsh's test, compared with control group. [c]One mouse died before randomization. [d]Mice's feces were sorted out of the wood-chip bedding, thus mice's fecal weight data are inaccurate.

**Table 4** Effect of feeding various pluronics on azoxymethane-induced aberrant crypt foci (ACF) and fecal values in F344 rats

| Group[a] | *n* | ACF/ colon | Crypts/ ACF | Large ACF > 3 crypts | Fecal weight (g/d) | Water in fresh feces (%) | Rat body weight (g) |
|---|---|---|---|---|---|---|---|
| Control | 10 | 72 ± 21[b] | 2.2 ± 0.2 | 5.6 ± 2.3 | 0.9 ± 0.3 | 39 ± 5 | 167 ± 5 |
| Pluronic F68 | 10 | 1.3 ± 2.1* | 1.9 ± 0.3 | 0.1 ± 0.3* | 1.7 ± 0.7+ | 65 ± 2* | 157 ± 5* |
| Pluronic F127 | 4 | 35 ± 12* | 2.3 ± 0.2 | 4.5 ± 3.8 | 1.8 ± 0.2 | 54 ± 3* | 166 ± 9 |
| Pluronic P85 | 4 | 68 ± 38 | 1.7 ± 0.1+ | 1.8 ± 1.3* | 1.0 ± 0.3 | 43 ± 1 | 158 ± 9 |
| Pluronic NP30 | 4 | 45 ± 04 | 1.9 ± 0.2 | 1.8 ± 1.0* | 1.4 ± 0.3 | 52 ± 2* | 158 ± 4 |
| Pluronic L61 | 4 | 48 ± 10 | 1.8 ± 0.3 + | 1.3 ± 1.5* | 1.1 ± 0.3 | ND | 151 ± 6* |
| Pluronic L64 | 4 | 51 ± 17 | 1.8 ± 0.1 + | 0.8 ± 1.0* | 1.0 ± 0.1 | ND | 158 ± 12 |
| ANOVA *P* | | <0.0001 | 0.003 | <0.0001 | 0.1 | <0.0001 | 0.003 |

[a]Pluronics were included at 5% (w/w) in the AIN 76 diet given to rats for 30 days, starting 7 days after a single azoxymethane injection. Pluronics L61 and L64 were included at 5% in the diet for one week only because the rats did not eat it.
[b]Mean ± SD.* $P < 0.01$ and + $P < 0.05$ by Dunnet's test, compared with control group. ND: Not done, because rats were fed the control diet before sacrifice.

blocks (Schmolka, 1994). 5 pluronics, PEG, and 3 other PEG-like agents with a hydrophobe moiety, were tested here (Table 1). Among the 9 agents we have tested, only the most water-soluble, PEG and PLU, were very potent against ACF (Table 1). The mechanism by which PLU decreases colon carcinogenesis is not known. Two mechanisms could be considered: healing of cell membranes, and dilution of gut content. (i) PLU markedly reduces cell loss and improves the growth of cells in culture (Swim and Parker, 1960), possibly because it strengthens the cell membranes (Zhang et al, 1992). The polyoxypropylene hydrophobe, not present in PEG, adheres to the hydrophobic area of the damaged cell membrane (Schmolka, 1994). PLU can thus plug holes, or facilitate membrane resealing (AlRubeai et al, 1993; Togo et al, 1999). Since cell wounding occurs to superficial cells in the lower gut (McNeil and Ito, 1989), we speculate that this healing effect might reduce crypt proliferation or block ACF growth. (ii) PLU, like PEG, is an osmotic laxative that increases fecal bulk and stools moisture, which dilutes bile acids (Corpet and Parnaud, 1999). However, many PEG-like agents that did not suppress ACF, increased fecal bulk as much as PLU (Tables 2 and 4). Moreover, PLU markedly decreased the ACF number in mice, without increasing the fecal bulk. We thus do not think that the dilution of gut content is the major mechanism of chemoprevention by PLU.

In conclusion, dietary PLU strikingly suppressed azoxymethane-induced preneoplastic lesions in the colon of rodents. PLU is not absorbed, and its per os toxicity is very low. PLU has a long and safe history of clinical applications, and has been given i.v. to several hundred patients (Schmolka, 1994). Based on body area, the protective dose in rats would translate to 20–40 g $d^{-1}$ in humans. In France, PLU is used as a common mild laxative at a dose of 6 g $d^{-1}$. PLU treatment can thus be associated with unpleasant side-effect. If long-term animal studies show that PLU can prevent macroscopic tumours, we think that its preventive features might be tested in humans at risk for colon cancer.

### ACKNOWLEDGEMENTS

This investigation was funded by the DGER and INRA of the Ministère de l'Agriculture, France. GP was supported by a studentship from the Ligue Nationale contre le Cancer, Gers, France.

### REFERENCES

AlRubeai M, Emery AN, Chalder S and Goldman MH (1993) A flow cytometric study of hydrodynamic damage to mammalian cells. *J Biotechnol* **31**: 161–177

 *© 2001 Cancer Research Campaign*

Bird RP (1987) Observation and quantification of aberrant crypts in murine colon treated with a colon carcinogen: preliminary findings. *Cancer Lett* **37**: 147–151

Caderni G, Giannini A, Lancioni L, Luceri C, Biggeri A, Dolara P (1995) Characterisation of aberrant crypt foci in carcinogen-treated rats: association with intestinal carcinogenesis. *Brit J Cancer* **71**: 763–769

Corpet DE and Parnaud G (1999) Polyethylene-glycol, a potent suppressor of azoxymethane-induced aberrant crypt foci in rats. *Carcinogenesis* **20**: 915–918

Corpet DE, Parnaud G, Delverdier M, Peiffer G, Taché S (2000) Consistent and fast inhibition of colon carcinogenesis by polyethylene-glycol, in mice and rats given various carcinogens. *Cancer Res* **60**: 3160–3164

Lentz BR (1994) Polymer-induced membrane fusion: potential mechanism and relation to cell fusion events. *Chem Phys Lipids* **73**: 91–106

McNeil PL and Ito S (1989) Gastrointestinal cell plasma membrane wounding and resealing in vivo. *Gastroenterology* **96**: 1238–1248

Parnaud G, Tache S, Peiffer G and Corpet DE (1999) Polyethylene-glycol suppresses colon cancer and causes dose-dependent regression of azoxymethane-induced aberrant crypt foci in rats. *Cancer Res* **59**: 5143–5147

Schmolka IR (1994) Physical basis for poloxamer interactions. *Ann NY Acad Sci* **720**: 92–97

Steele VE, Boone CW, Lubet RA, Crowell JA, Holmes CA, Sigman CC and Kelloff GJ (1998) Preclinical drug development paradigms for chemopreventives. *Hematol Oncol Clin North Am* **12**: 943–961

Swim HE and Parker RF (1960) Effect of Pluronic F68 on growth of fibroblasts in suspension on rotary shaker. *Proc Soc Exp Biol Med* **103**: 252–253

Togo T, Alderton JM, Bi GQ and Steinhardt RA (1999) The mechanism of facilitated cell membrane resealing. *J Cell Sci* **112**: 719–731

UKCCCR (1998) Guidelines for the welfare of animals in experimental neoplasia (2nd ed.) *Brit J Cancer* **77**: 1–10

Zhang Z, AlRubeai M and Thomas CR (1992) Effect of pluronic F-68 on the mechanical properties of mammalian cells. *Enzyme Microb Technol* **14**: 980–983

© 2001 Cancer Research Campaign

